# Unexpected Zoonotic and Hybrid Schistosome Egg Excretion Patterns, Malawi, 2024

**DOI:** 10.3201/eid3105.241757

**Published:** 2025-05

**Authors:** Angus M. O’Ferrall, Sekeleghe A. Kayuni, Lucas J. Cunningham, Peter Makaula, John Archer, Adam P. Roberts, Janelisa Musaya, J. Russell Stothard

**Affiliations:** Liverpool School of Tropical Medicine, Liverpool, UK (A.M. O’Ferrall, S.A. Kayuni, L.J. Cunningham, J. Archer, A.P. Roberts, J.R. Stothard); Malawi-Liverpool-Wellcome Trust Clinical Research Programme, Queen Elizabeth Central Hospital, Blantyre, Malawi (S.A. Kayuni, P. Makaula, J. Musaya); School of Medicine and Oral Health, Kamuzu University of Health Sciences, Blantyre (S.A. Kayuni, J. Musaya); Wolfson Wellcome Biomedical Laboratories, Natural History Museum, London, UK (J. Archer)

**Keywords:** schistosomiasis, parasites, zoonoses, Schistosoma haematobium, Schistosoma mansoni, Schistosoma mattheei, hybridization, One Health

## Abstract

Two exemplary cases of mixed urogenital and intestinal schistosomiasis in Malawi show hybridizations of *Schistosoma mattheei* with *S. haematobium* and *S. mansoni*, indicating newly emerging genetic diversity. Complex egg excretion patterns in feces expose current diagnostic gaps and alert to future sampling needs for effective surveillance of zoonotic schistosomiasis.

Schistosomiasis is a waterborne, parasitic disease transmitted by several species of *Bulinus* and *Biomphalaria*, two distinct freshwater snail genera common across sub-Saharan Africa (*1*). In sub-Saharan Africa, *Schistosoma haematobium* is the predominant cause of urogenital schistosomiasis, and *S. mansoni* is the predominant cause of intestinal schistosomiasis (*1*). *S. haematobium* is endemic in Malawi, where infections with zoonotic and hybrid species from the *S. haematobium* group (*S. mattheei* and *S. haematobium* × *S. mattheei*) have also been detected in humans (*2*,*3*). *S. mattheei* is considered a livestock-infecting schistosome that causes intestinal disease (*4*); however, excretion of ova from humans infected with *S. mattheei* and associated *S. haematobium* group hybrids reportedly has occurred through the urogenital tract (*2*,*3*). Meanwhile, *Biomphalaria* freshwater snails were first detected along the southern shores of Lake Malawi in 2017 (*5*). Since then, autochthonous *S. mansoni* transmission and intestinal schistosomiasis outbreaks have been confirmed in Mangochi District, Malawi (*5*,*6*).

To clarify *S. haematobium* group hybridization dynamics, we conducted a longitudinal cohort study in southern Malawi. The College of Medicine Research Ethics Committee, Malawi (approval no. P.08/21/3381, https://www.ncst.mw) and the Liverpool School of Tropical Medicine Research Ethics Committee, United Kingdom (approval no. 22-028, https://www.lstmed.ac.uk/research/research-integrity/research-ethics-committee) provided ethics approval. This study also tracked *S. mansoni* prevalence in a community cohort recruited from Samama Village, Mangochi District (Appendix Figure 1), where the outbreak of intestinal schistosomiasis was initially reported (*5*,*6*).

In June 2024, we determined *S. mansoni* prevalence in Mangochi District to be 14.8% (165/1,116) using point-of-care urine circulating cathodic antigen cassette tests (POC-CCA; ICT International, https://ictinternational.com), and considered trace results positive. Those results represented the lowest reported *S. mansoni* prevalence in Samama Village since it emerged in 2017 (*5*–*7*). However, we observed numerous atypical schistosome ova within feces provided by 2 POC-CCA–positive participants upon Kato-Katz examination (https://microbeonline.com/kato-katz-technique-principle-procedure-results) (Figure). Patient X, a 10-year-old girl, and patient Y, a 19-year-old man (Table), both received treatment with praziquantel from a study-affiliated clinician. The unexpected morphologic diversity raised concerns about underreporting of intestinal schistosomiasis being caused by species other than *S. mansoni*, prompting closer molecular analysis for robust speciation that cannot be achieved by microscopy.

**Figure:**
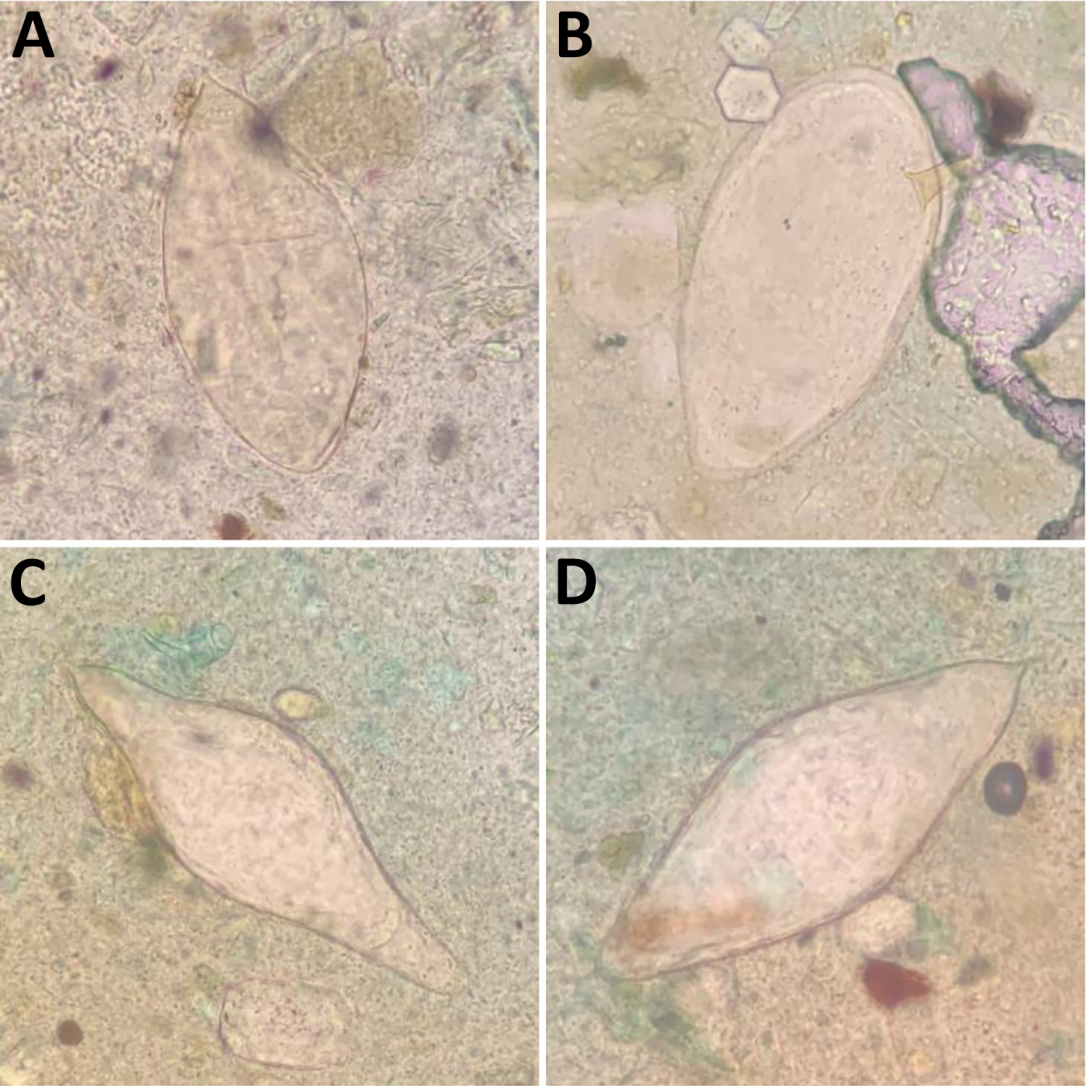
Morphologic *Schistosoma* spp. identification from 2 patient’s feces samples in an investigation of unexpected zoonotic and hybrid schistosome egg excretion patterns, Malawi, 2024. A) Typical *S. haematobium* (length 130 μm); B) typical *S. mansoni* (length 150 μm); C) atypical terminal-spined egg (length 188 μm); D) atypical terminal-spined egg (length 154 μm). Samples were stained with methylene blue glycerol solution using the Kato-Katz method (https://microbeonline.com/kato-katz-technique-principle-procedure-results). Atypical morphology (C,D) prompted closer molecular analysis for speciation, which revealed *S. haematobium* × *S. mattheei* hybrids.

**Table:** Summary of diagnostic information for patient X and patient Y from unexpected zoonotic and hybrid schistosome egg excretion patterns, Malawi, 2024*

Diagnostic test	Patient X	Patient Y
Urinary diagnostics		
Eggs/10 mL urine (no. typed)†	>50 (71)	30 (50)
Microhematuria	Large	Large
Visible hematuria	Yes	Yes
Proteinuria	>2,000 mg/dL	>2,000 mg/dL
Turbid urine	Yes	Yes
POC-CCA	Positive	Positive
Fecal diagnostics		
Eggs/g feces (no. typed)†	84 (60)	240 (34)
Fecal occult blood	Negative	Positive

*POC-CCA, point-of-care urine circulating cathodic antigen cassette tests. †Miracidia typing.

We obtained hatched miracidia from the urine and feces of patients X and Y by using Pitchford-Visser filtration and collected and preserved individual miracidia on Whatman Flinders Technology Associates cards (GE Healthcare Life Sciences, https://www.gehealthcare.com/products/life-sciences), according to standard protocols (*8*). To identify the schistosome larvae, we used a newly described 2-tube high-resolution melt real-time PCR assay on DNA extracted from individual preserved miracidia (*9*). To detect evidence of mixed ancestry or putative genetic introgression, we targeted both the nuclear DNA ribosomal internal transcribed spacer 2 locus and species-specific mitochondrial DNA (mtDNA) loci of 6 *Schistosoma* species: *S. bovis*, *S. curassoni*, *S. haematobium*, *S. mansoni*, *S. margrebowiei*, and *S. mattheei*. For *S. bovis*, *S. curassoni*, *S. haematobium*, and *S. mansoni* we targeted the tRNA lysine region; for *S. margrebowiei* the NADH dehydrogenase subunit 4 region; and for *S. mattheei*, the NADH dehydrogenase subunit 6 region.

Miracidia hatched from ova in the feces of patient X mostly typed as *S. haematobium* × *S. mattheei* hybrids (93.3%), whereas most miracidia hatched from the paired urine sample typed as pure *S. haematobium* (95.8%). Similarly, atypical zoonotic and hybrid schistosome species ova from patient Y were predominantly in feces (Appendix Figure 2). Of the 59 *S. haematobium* × *S. mattheei* hybrid miracidia typed from feces, high-resolution melt profiles indicated that mtDNA (maternal) was inherited from *S. mattheei* in 58 miracidia, although *S. haematobium* mtDNA was detected in the remaining *S. haematobium* × *S. mattheei* miracidia. From patient Y, 1 miracidium showed mixed ancestry between *S. mansoni* and *S. mattheei*, with discordance between mtDNA and nuclear DNA profiles (Appendix Figure 3). Although adult worm pairings between distantly related species usually result in the production of parthenogenetic eggs, previous experimental pairings of *S. mansoni* and *S. mattheei* resulted in the production of eggs viable to the third generation (*10*).

Our findings not only provide evidence of complex hybridization events in the natural setting among *S. haematobium*, *S. mansoni*, and *S. mattheei* but also highlight the greater relative abundance of zoonotic and hybrid schistosome species ova in feces compared with paired urine samples. That observation suggests that *S. mattheei* and associated hybrids, previously linked to urogenital schistosomiasis in humans, may dominate in intestinal infections by migrating to the intestinal mesenteries, just as *S. mattheei* likely does in other mammalian hosts where it causes rectal schistosomiasis (*4*). POC-CCA tests were not designed to detect zoonotic schistosomiasis; although we acknowledge that patients X and Y were both POC-CCA positive, those results do not assure the ability of POC-CCA tests to detect *S. haematobium* group intestinal infections because we did not perform detailed inspection of feces from POC-CCA–negative participants in the field. 

In summary, we detected *S. haematobium* × *S. mattheei* hybrid ova from 2 patients in Malawi. Further fecal sampling and molecular testing with species-specific TaqMan probe assays will be essential for monitoring intestinal schistosomiasis in coendemic areas where zoonotic transmission could occur.

Appendix Additional information for unexpected zoonotic and hybrid schistosome egg excretion patterns, Malawi, 2024.
