# Supplementary material for: Unexpected Zoonotic and Hybrid Schistosome Egg Excretion Patterns, Malawi, 2024
Source: Emerg Infect Dis. 2025 May;31(5):1042–5. doi: 10.3201/eid3105.241757 (PMC12044234; doi:10.3201/eid3105.241757)
Supplement: Appendix — Additional information for unexpected zoonotic and hybrid schistosome egg excretion patterns, Malawi, 2024. [file 24-1757-Techapp-s1.pdf]

Article DOI: https://doi.org/10.3201/eid3105.241757

EID cannot ensure accessibility for supplementary materials supplied by authors. Readers who have difficulty accessing supplementary content should contact the authors for assistance.

Unexpected Zoonotic and Hybrid Schistosome Egg Excretion Patterns, Malawi, 2024

Appendix

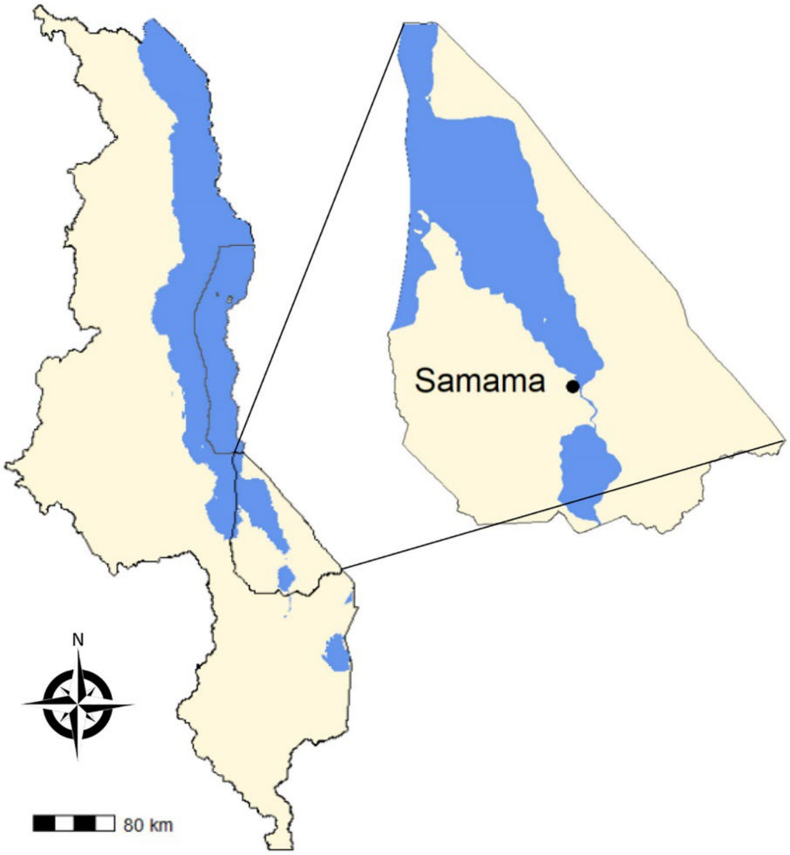

Appendix Figure 1. Sampling location: Samama Village, Mangochi District, Malawi.

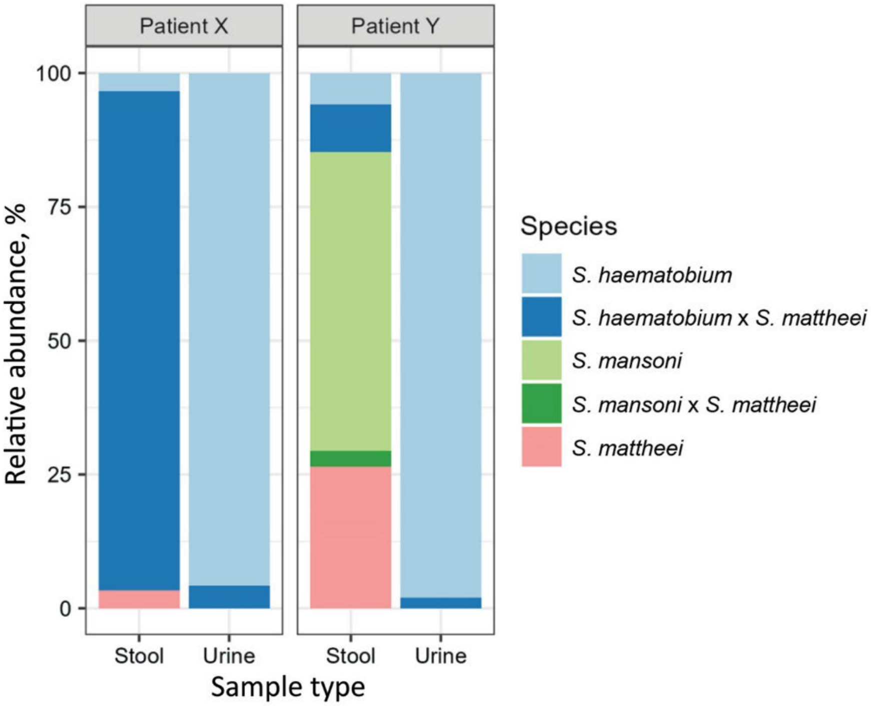

Appendix Figure 2. Relative abundance of different *Schistosoma* species miracidia hatched from feces and urine from patient X and patient Y.

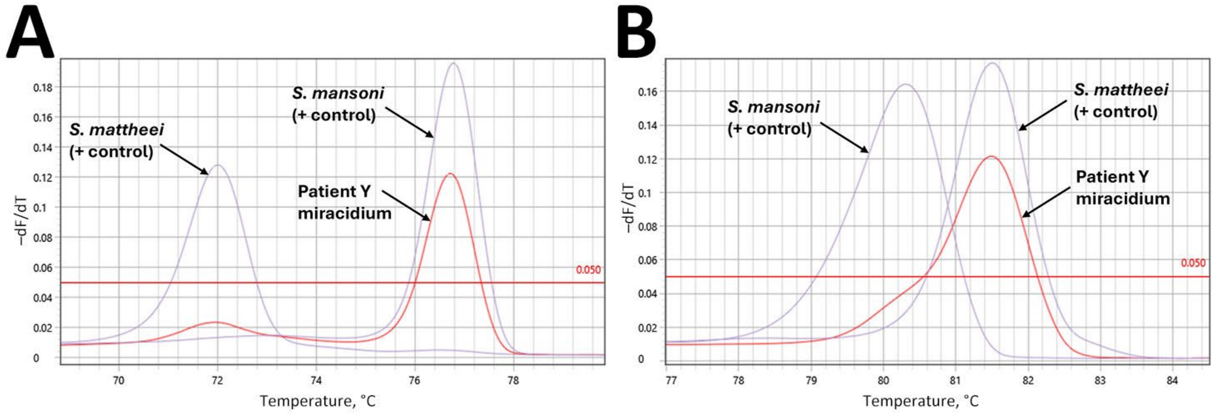

Appendix Figure 3. mtDNA and nDNA high-resolution melt real-time PCR curves indicating the excretion of an *S. mansoni* × *S. mattheei* hybrid egg in the feces of patient Y.
